# Supplementary material for: Computationally designed GPCR quaternary structures bias signaling pathway activation
Source: Nat Commun. 2022 Nov 11;13:6826. doi: 10.1038/s41467-022-34382-7 (PMC9652377; doi:10.1038/s41467-022-34382-7)
Supplement: Supplementary file 1 — Supplementary Information [file 41467_2022_34382_MOESM1_ESM.pdf]

Computationally designed GPCR quaternary structures bias signaling pathway activation

Justine S. Paradis, Xiang Feng, Brigitte Murat, Robert E. Jefferson, Badr Sokrat, Martina Szpakowska, Mireille Hogue, Nick D. Bergkamp, Franziska M. Heydenreich, Martine J. Smit, Andy Chevigne, Michel Bouvier, Patrick Barth

*Correspondences should be addressed to: Michel Bouvier, email: michel.bouvier@umontreal.ca and Patrick Barth, e-mail: patrick.barth@epfl.ch

Supplementary Table 1. Distribution of closed and open CXCR4 dimer conformations. The dimer interface energy in Rosetta Energy Units are reported for the best open-dimer or closed-dimer conformations adopted by each CXCR4 variant modeled in the inactive or active state.

	INACTIVE STATE		ACTIVE STATE	
	Dimer Interface Energy		Dimer Interface Energy	
	Open-dimer	Closed-dimer	Open-Dimer	Closed-dimer
WT	-24.28	-18.38	-25.39	-21.06
$N192_{ECL2}W$	-22.67	-22.16	-19.52	-25.09
$W195_{5.34}L$	-23.59	-21.53	-21.3	-26.07
$L194_{5.33}R$	-26.68	-20.83	-24.7	-20.75

Supplementary Table 2. Relationship between calculated dimerization propensity and dimerization BRET signals measured for CXCR4 variants. The predicted dimerization propensities of the CXCR4 variants relative to the WT receptor are calculated from the energies of the dimer conformations in the inactive and active states (see Methods). The net dimerization BRET signals (**see Fig. 3**) normalized to WT are provided for comparison.

	INACTIVE STATE		ACTIVE STATE	
	Predicted dimerization propensity	Constitutive net BRET	Predicted dimerization propensity	Agonist induced net BRET
WT	1.00	1.00	1.00	1.00
$N192_{ECL2}W$	0.42	0.57	0.76	0.69
$W195_{5.34}L$	0.65	0.81	1.76	1.22
$L194_{5.33}R$	7.57	0.95	0.56	0.88

Supplementary Table 3. Distribution of open and wide-open μOR dimer conformations. The dimer interface energy in Rosetta Energy Units are reported for the best open dimer or wide-open dimer conformations adopted by each μOR variant modeled in the active state.

	ACTIVE STATE					
	Dimer Interface Energy		Beta arrestin binding energy		Go binding energy	
	Open-dimer	Wide-open dimer	Open-dimer	Wide-open dimer	Open-dimer	Wide-open dimer
WT	-27.9	-22.7	-9	-6.3	-8.7	-8.3
W230$_{5.34}$A	-26.7	-23.3	-8.1	-6.3	-8.4	-8.4

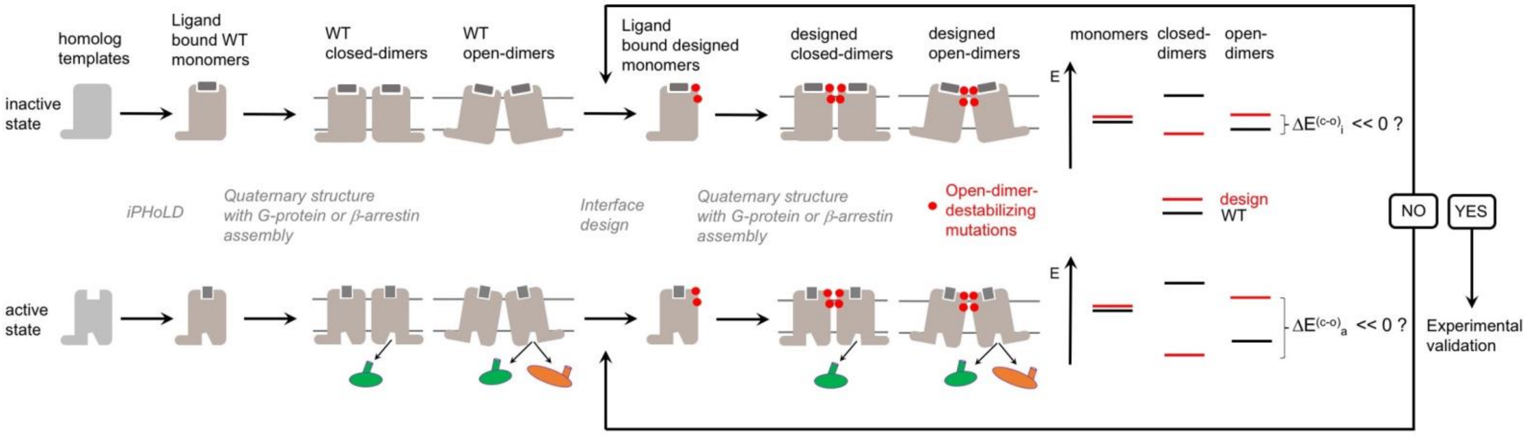

Supplementary Fig. 1. Workflow of the QUESTS computational approach. Starting from homolog templates, ligand-bound GPCR inactive and active state monomers are generated using iPHoLD[1]. Monomers are assembled into GPCR dimers and into complex with G-protein or β-arrestin by flexible docking. Loop and residue motifs are designed at the predicted dimer interface to destabilize (in this example) or stabilize selectively a specific dimer conformation (e.g. open-dimer). Designed monomers are assembled into GPCR dimers and into complex with G-proteins or β-arrestin to assess the shift in dimer conformations distribution (calculated as the energy difference between the closed and open designed structures, $\Delta E^{(c\text{-}o)}{}_{i}$) and the associated functional (i.e. G-protein versus β-arrestin binding) shift. The design-quaternary structure assembly cycle is repeated until a significant shift is achieved, i.e. $\Delta E^{(c\text{-}o)}{}_{i}$ and $\Delta E^{(c\text{-}o)}{}_{a}$ << 0 providing monomer stability is not affected significantly.

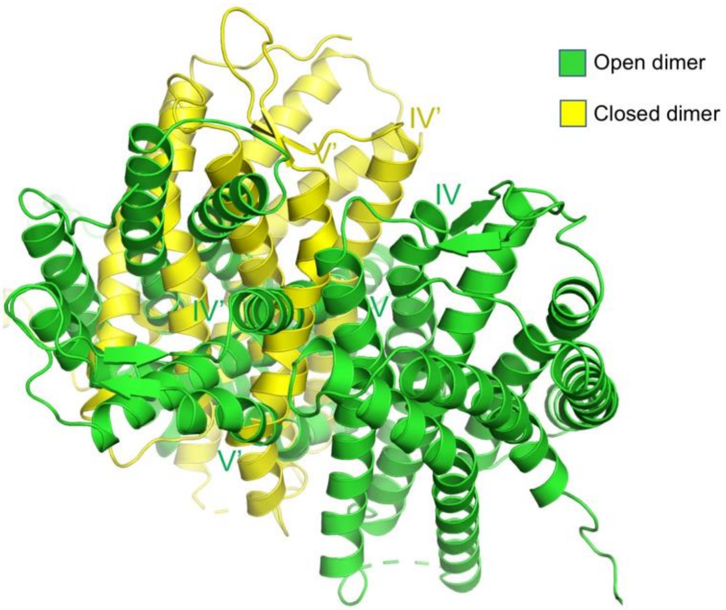

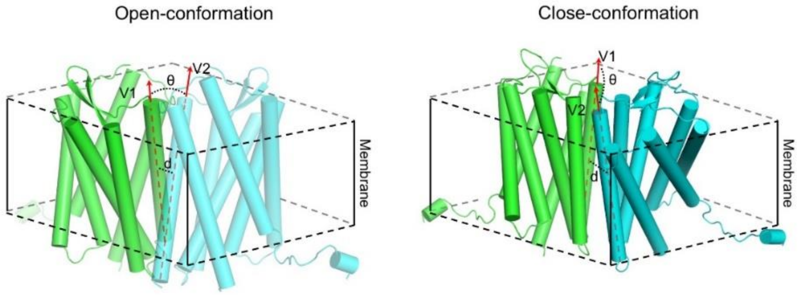

	INACTIVE STATE				ACTIVE STATE			
	Angle (deg)		Distance (Angstrom)		Angle (deg)		Distance (Angstrom)	
	Open-dimer	Closed-dimer	Open-dimer	Closed-dimer	Open-dimer	Closed-dimer	Open-dimer	Closed-dimer
WT	60.62	-8.86	14.24	19.71	51.54	26.59	14.45	20.62
N192W	55.07	-10.90	13.92	22.09	59.74	29.14	12.61	21.57
W195L	74.18	1.35	13.10	21.83	55.82	35.03	12.27	21.43
L194R	58.49	-17.45	15.46	23.66	50.35	23.72	12.72	21.44

Supplementary Fig. 2. Geometrical comparison between the open and closed CXCR4 dimer conformations. Top. Superposition between closed and open dimer conformations (the aligned monomer of the closed form is omitted for clarity). TM helices IV and V are labelled in each protomer. The Cα rmsd between the open-dimer inactive state model and the antagonist bound X-ray structure (3odu) is 3.5 Å over the entire dimer structure. **Bottom.** The interhelical angle and distance between the TM helix 5 of each monomer are used to characterize the dimer conformation. A vector is fitted to the Cα coordinates of each TM helix 5. The angle and mid-point distance between the two vectors are measured. Two representative models of the open-dimer ($\theta = 53^{\circ}$ and d = 13.6 Å, left) and closed-dimer conformation ($\theta = -8.2^{\circ}$ and d = 21.4 Å, right) are shown.

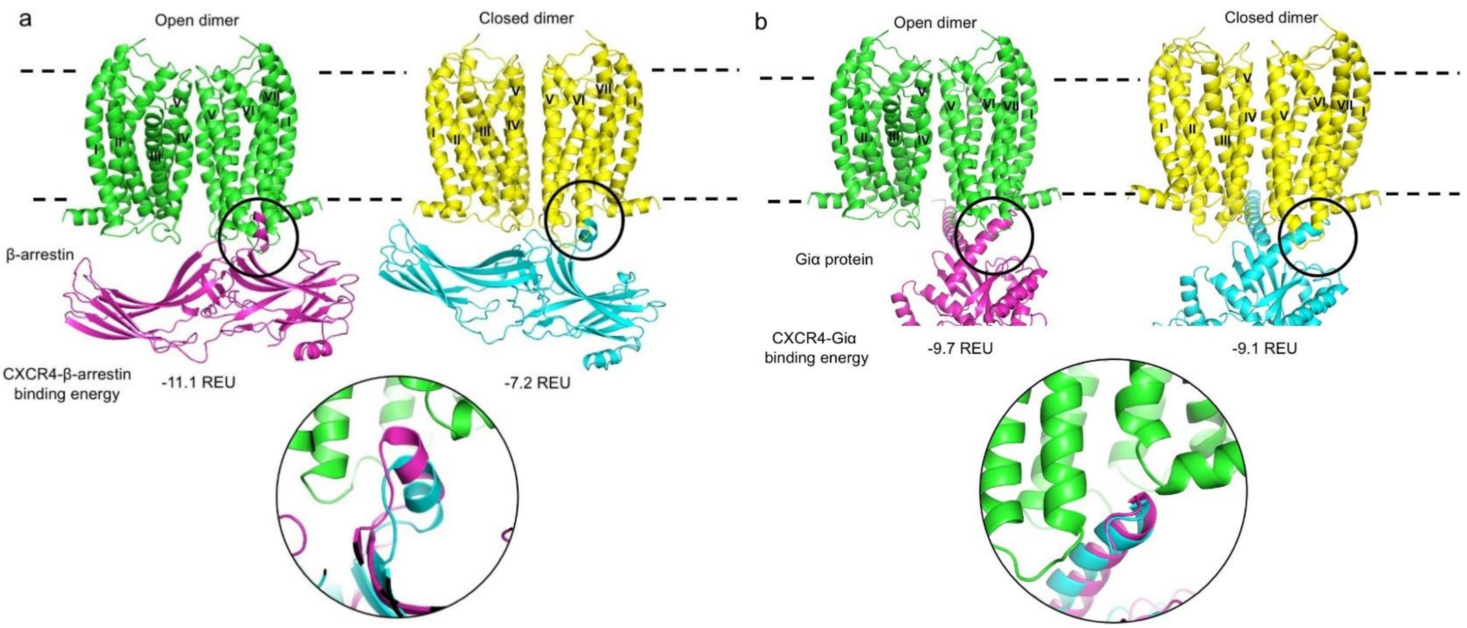

Supplementary Fig. 3. CXCR4 strongly interacts with β-arrestin in the open-dimer conformation only. Representative lowest energy open and closed dimer conformations of CXCR4 WT in the active state bound to β-arrestin (a) and Gi (b). The structures with the lowest binding energy between the receptor and each effector are shown with a zoomed view of the main binding interface. The binding energies between the CXCR4 conformations and β-arrestin or Gi are provided in Rosetta Energy Units (REU). The closed-dimer conformation prevents optimal binding of the β-arrestin's finger loop with the CXCR4 monomer intracellular binding groove. TM helices are labelled on each CXCR4 protomer.

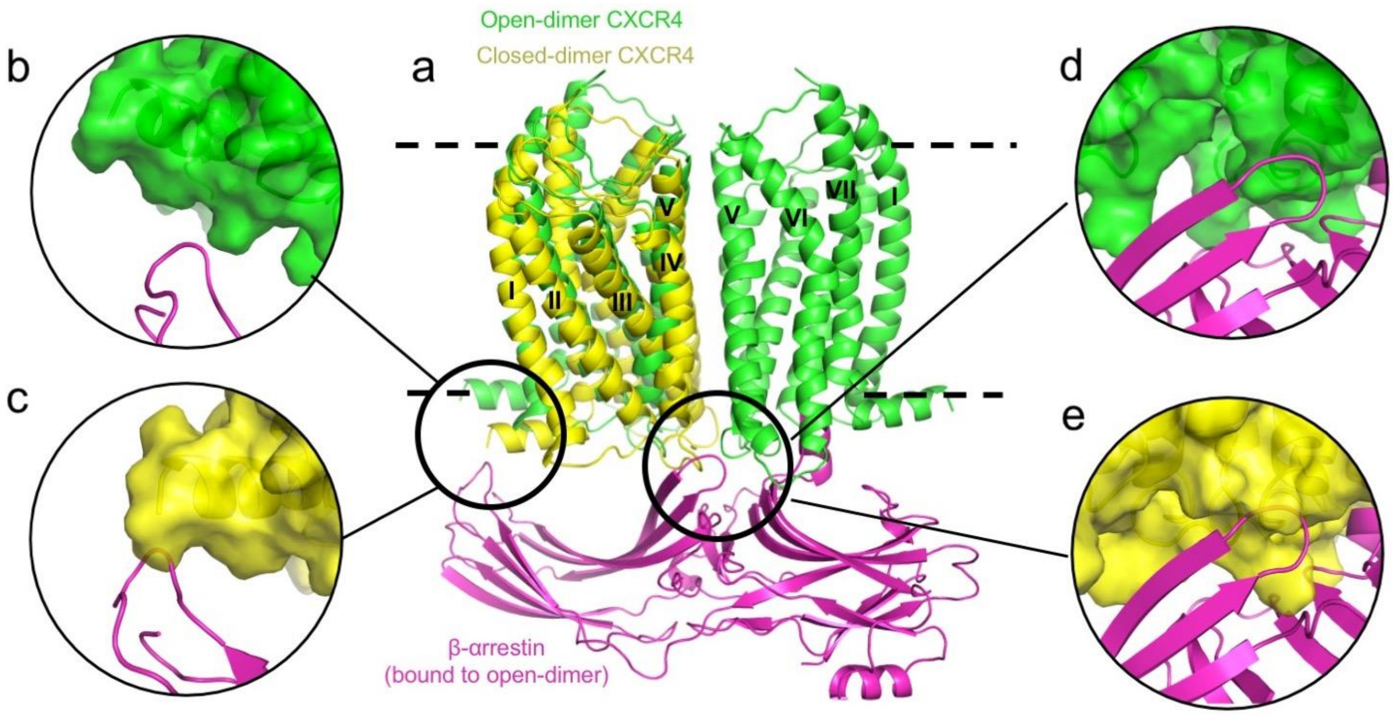

Supplementary Fig. 4. Steric hindrance prevents CXCR4 in the closed dimer conformation to strongly interact with β-arrestin. a. The closed-dimer conformation is aligned to the β-arrestin-bound open-dimer receptor complex by superimposing one CXCR4 monomer. Open- and closed- dimer conformations are colored in green and yellow, respectively. The docked β-arrestin is colored in magenta. The two insets highlight regions of close contacts between the β-arrestin and the open-dimer CXCR4 (**b:** Helix 8 of CXCR4 monomer 2 with the C-tip of β-arrestin**, d:** ICL2 of CXCR4 monomer 1 with the C-loop of β-arrestin) that would be disrupted in the closed-dimer conformation because of major steric clashes between the β-arrestin and the receptor second monomer (**c, e**). TM helices are labelled on each CXCR4 protomer.

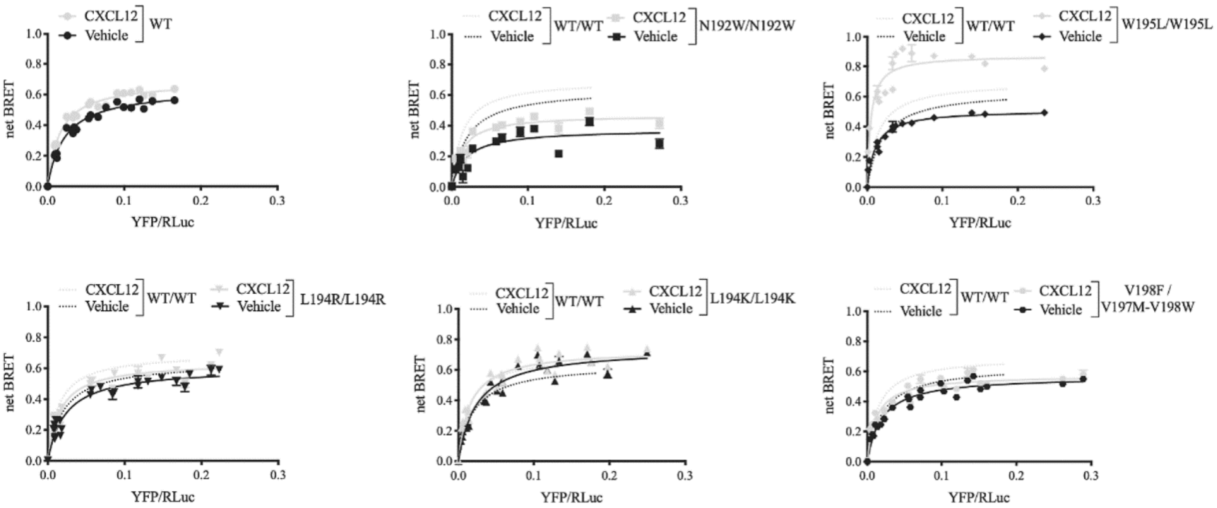

Supplementary Fig. 5. Titration curves of CXCR4 association. CXCR4 association BRET titration curves were performed in cells co-transfected with a constant amount of CXCR4-Rluc and increasing amounts of WT or mutant forms of CXCR4-YFP, as indicated. $BRET_{480\text{-}YFP}$ was measured after the addition of coel-h (10 min) and 200 nM CXCL12 or vehicle (15 min). The curves shown are derived from individual titration curves that are representative of three independent experiments. The error bars represent the mean ± SD derived from triplicate.

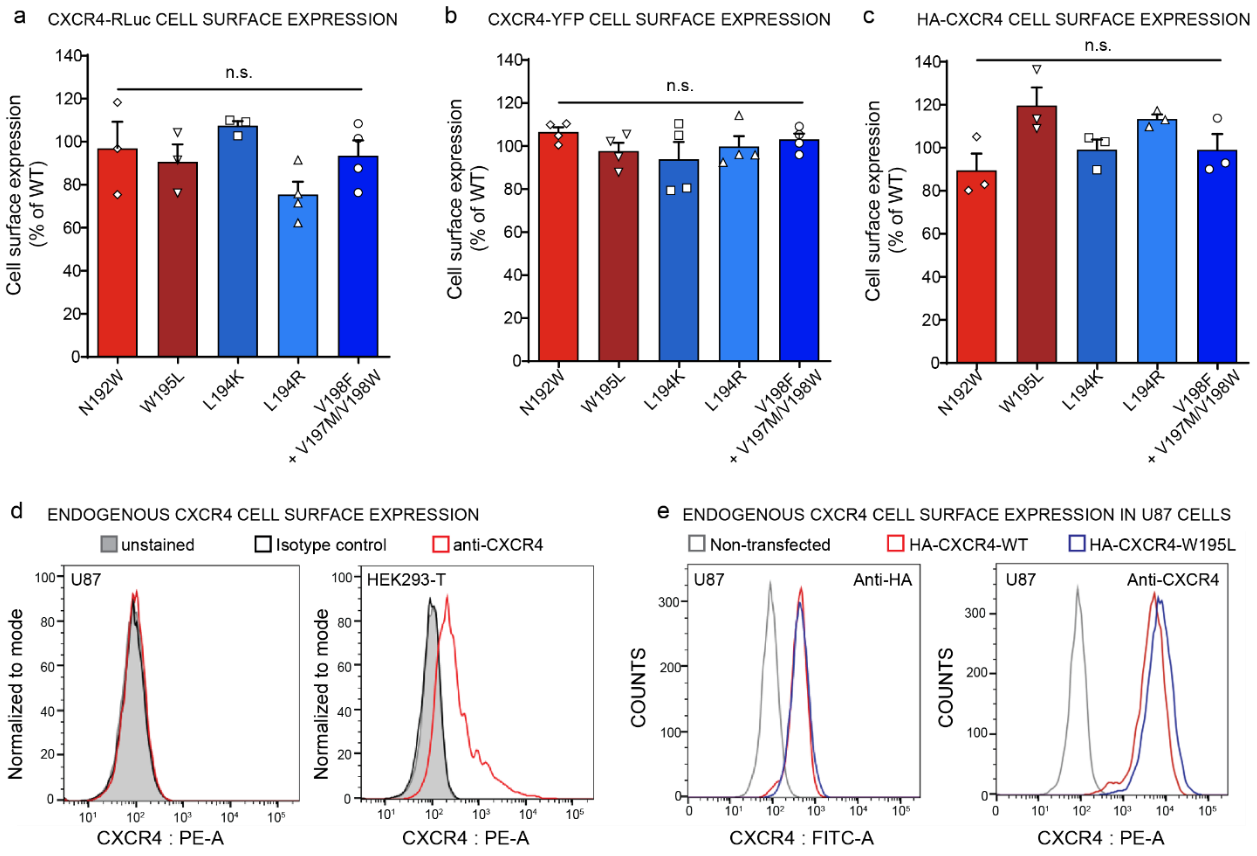

Supplementary Fig. 6. Cell surface expression of CXCR4 variants. a-c. Cell surface expression of Myc-CXCR4-RLuc (**a**), HA-CXCR4-YFP (**b**) and HA-CXCR4 (**c**), as well as their respective mutant forms, was determined by ELISA using an anti-Myc (**a**) or anti-HA (**b, c**) antibody. Data shown represent the mean ± SEM of at least three independent experiments. Statistical significance was assessed using a one-way ANOVA followed by Tukey's multiple comparison test: n.s. not significant p>0.05. **d.** Cell surface expression of endogenous WT CXCR4 in U87 versus HEK293-T cells was determined by Flow cytometry using an anti-CXCR4 antibody. **e.** Cell surface expression in U87 cells of exogenous HA-CXCR4, WT or W195$^{5.34}$L, was determined by Flow cytometry using an anti-CXCR4 (left) and HA antibody (right).

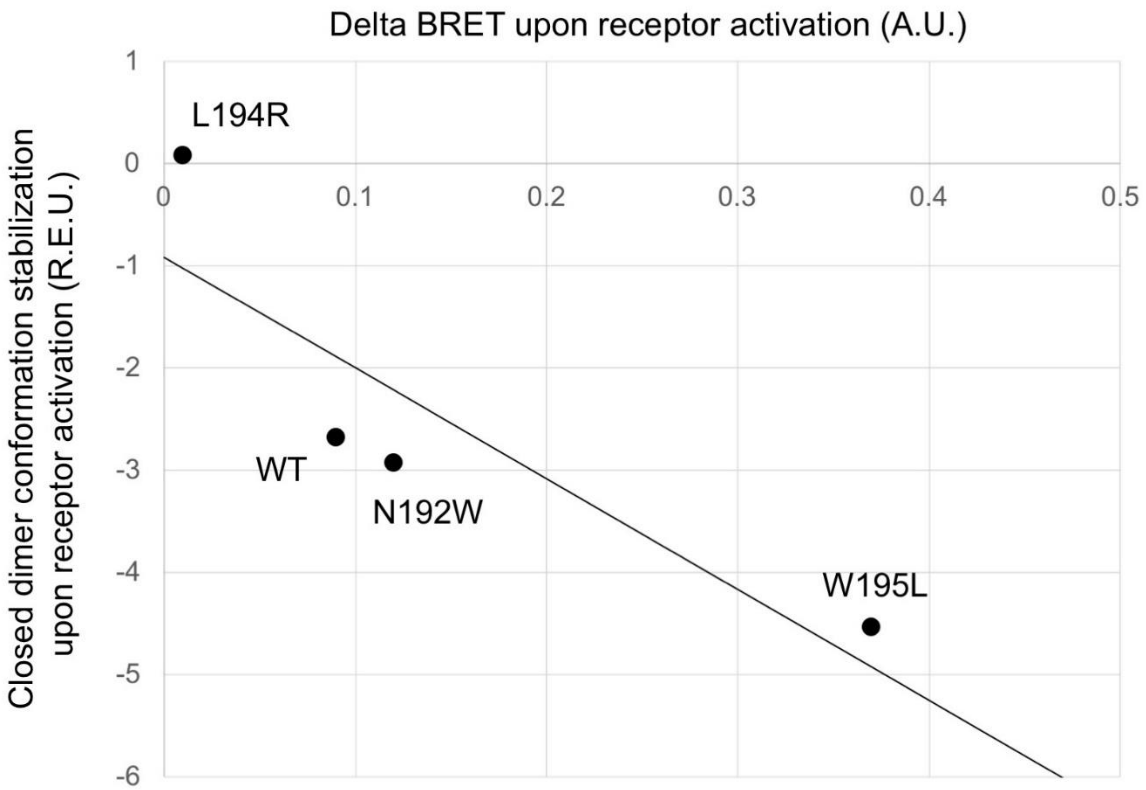

Supplementary Fig. 7. Correlation between closed dimer stabilization and BRET signal changes upon receptor activation.

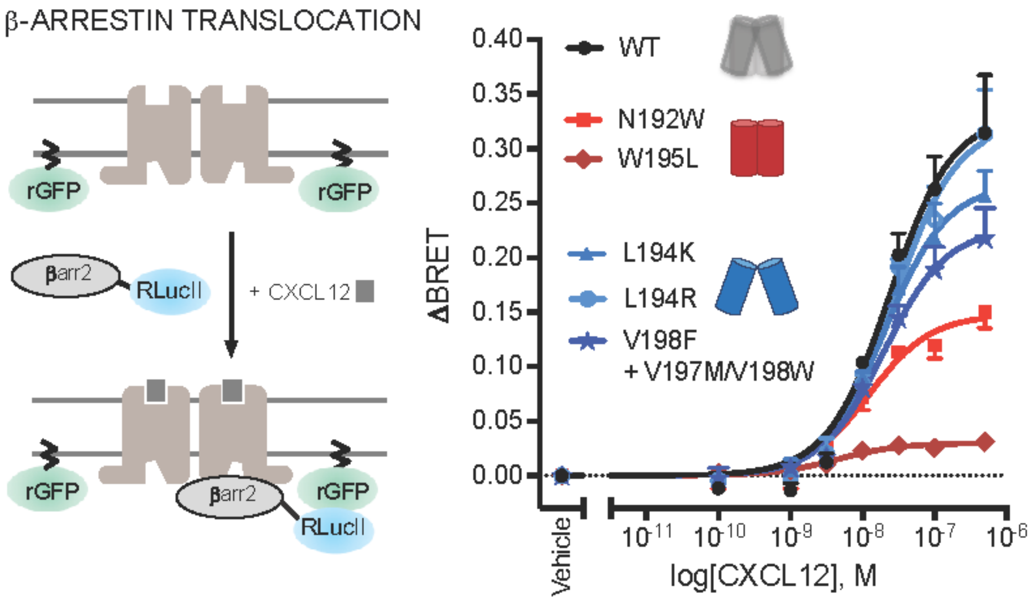

Supplementary Fig. 8. Translocation of β-arrestin2 to the plasma membrane. (Left) Schematic representation of the ebBRET-based assay used to follow agonist-induced βarr2 recruitment to the receptor by monitoring the interaction between βarr2-RLucII and rGFP-CAAX. (Right) Cell surface translocation of βarr2 was assessed by BRET400-GFP10 in HEK293T transfected with HA-CXCR4, WT or mutant as indicated, βarr2-RLucII and rGFP-CAAX. BRET400-rGFP between βarr2-RLucII and CAAX-rGFP was measured after the addition of coel-400a (5 min) and CXCL12 (15 min). Data shown represent the mean ± SEM of at least three independent experiments and are expressed as agonist-promoted BRET(ΔBRET). CXCR4 mutations predicted to stabilize the open-dimer or the closed-dimer conformation are annotated with a blue or red dimer symbol, respectively.

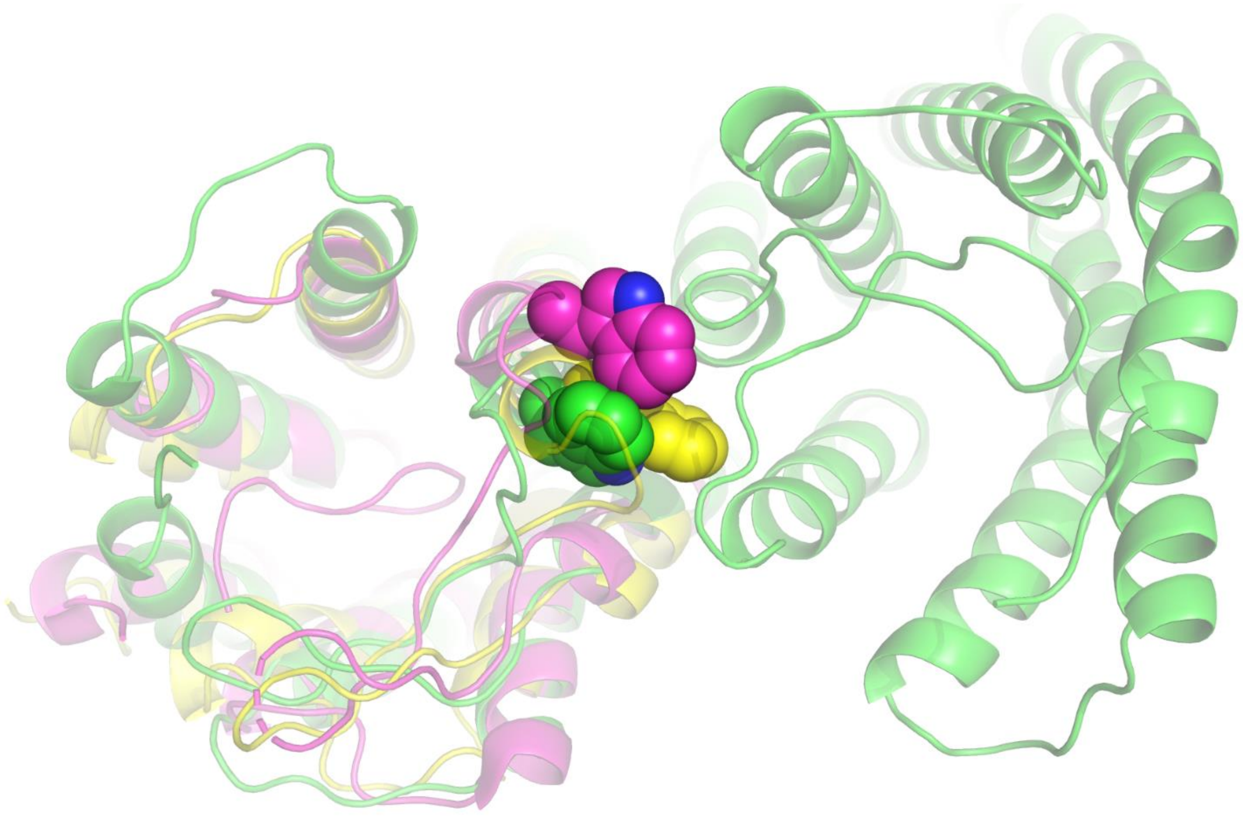

Supplementary Fig. 9. W5.34 in mOR and W5.33 in V2R point in a very similar direction than W5.34 in CXCR4. Superposition between the open dimer conformation of CXCR4 (green) and the mOR (yellow, pdbid: 6ddf) and V2R-Gs structures (magenta, pdbid: 7dw9). The sidechains of W5.34 / 5.33 are represented in sphere.

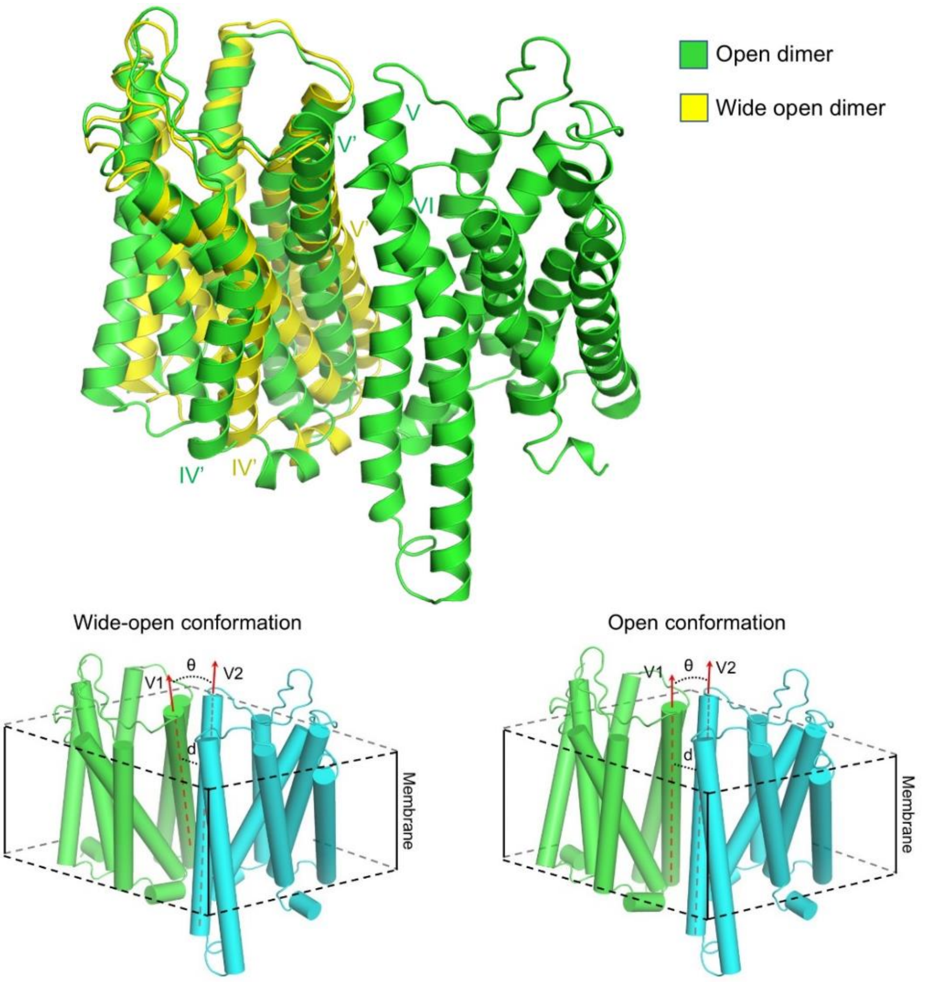

	ACTIVE STATE			
	Angle (deg)		Distance (Angstrom)	
	Open-dimer	Wide-open-dimer	Open-Dimer	Wide-open-dimer
WT	30.42	44.62	11.8	13.7
W5.34A	29.75	43.79	11.7	13.86

Supplementary Fig. 10. Geometrical comparison between the open and closed μOR dimer conformations. Top. Superposition between the Open and Wide-open dimer conformations (the aligned monomer of the Wide-open form is omitted for clarity). TM helices V and IV or VI are labelled in each protomer. **Bottom.** The interhelical angle and distance between the TM helix 5 of each monomer are used to characterize the dimer conformation. A vector is fitted to the Cα coordinates of each TM helix 5. The angle and mid-point distance between the two vectors are measured. Two representative μOR WT models of the Wide-open-dimer ($\theta = 45^{\circ}$ and d = 13.7Å, left) and Open-dimer conformation ($\theta = 30^{\circ}$ and d = 11.8Å, right) are shown.

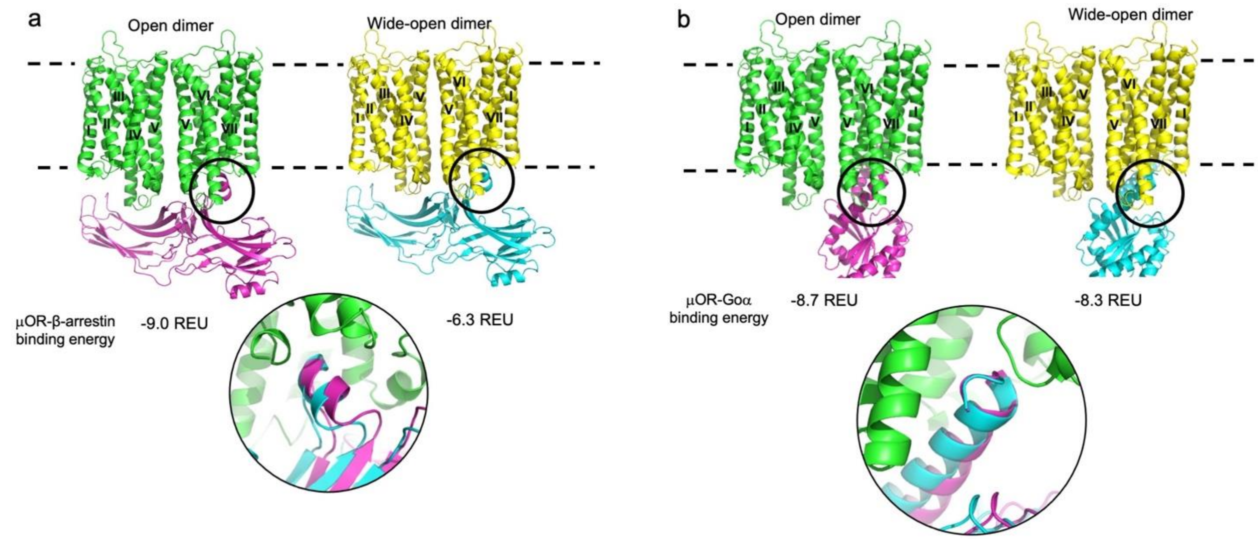

Supplementary Fig. 11. μOR strongly interacts with β-arrestin in the open-dimer conformation only. Representative lowest energy open and wide-open dimer conformations of μOR WT in the active state bound to β-arrestin (a) and Go (b). The structures with the lowest binding energy between the receptor and each effector are shown with a zoomed view of the main binding interface. The binding energies between the μOR conformations and β-arrestin or Go are provided in Rosetta Energy Units (REU). The closed-dimer conformation prevents optimal binding of the β-arrestin's finger loop with the μOR monomer intracellular binding groove. TM helices are labelled on each μOR protomer.

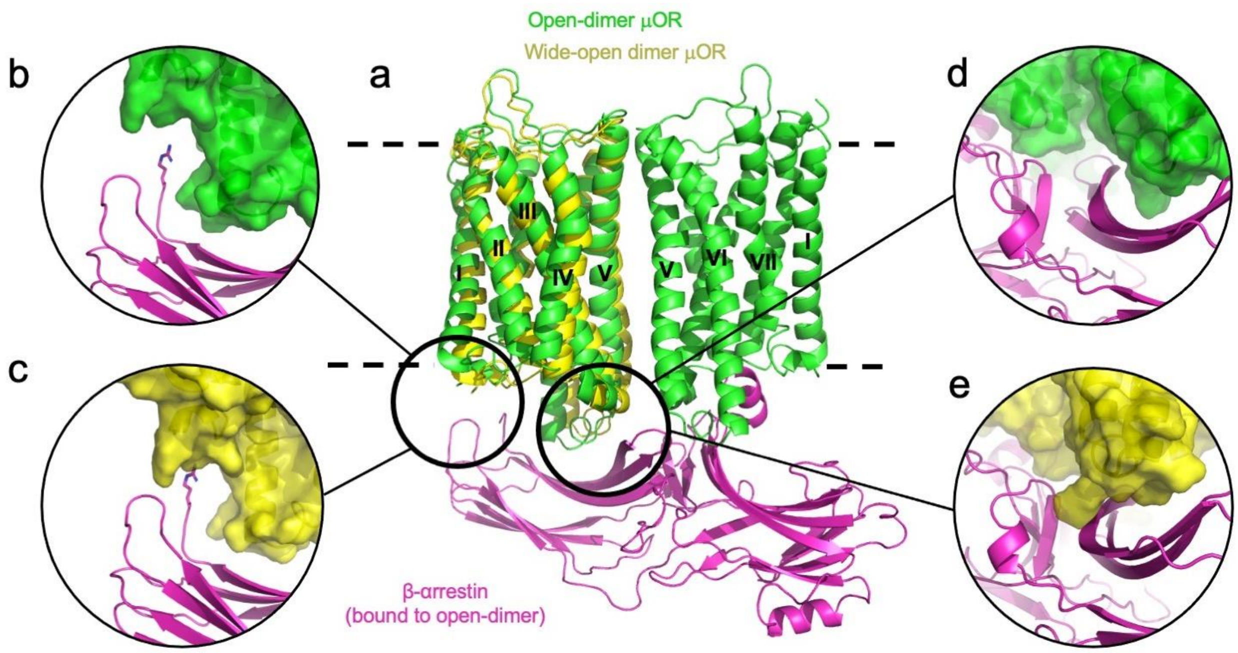

Supplementary Fig. 12. Steric hindrance prevents μOR in the closed dimer conformation to strongly interact with β-arrestin. a. The wide-open dimer conformation is aligned to the arrestin-bound open-dimer receptor complex by superimposing one μOR monomer. Open and wide-open dimer conformations are colored in green and yellow, respectively. The docked arrestin is colored in magenta. The two insets highlight regions of close contacts between the arrestin and the open-dimer mOR (**b:** Helix 7 of μOR monomer 2 with the C-tip of β-arrestin**, d:** ICL3 of μOR monomer 2 with the C-loop of β-arrestin) that would be disrupted in the wide-open dimer conformation because of major steric clashes between the arrestin and the receptor second monomer (**c, e**).

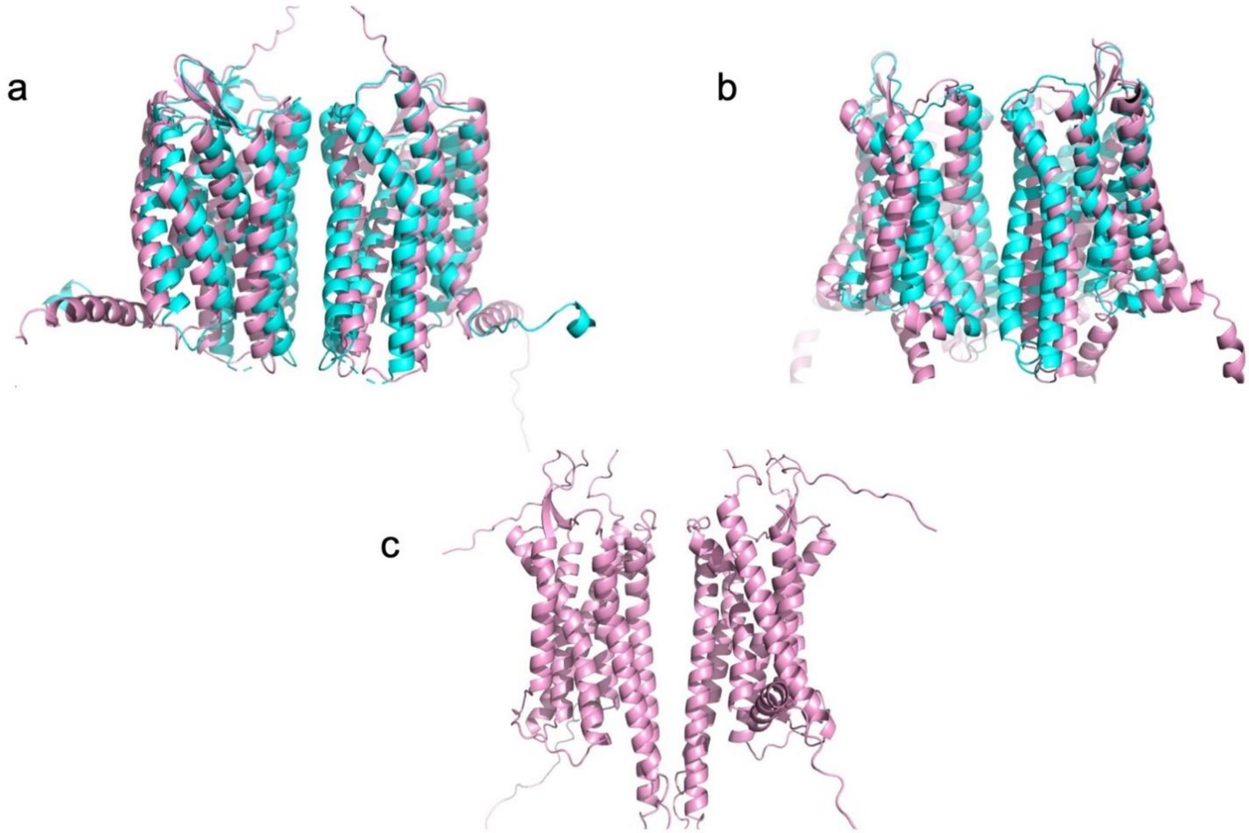

Supplementary Fig. 13. Comparison between AF2 and QUESTS receptor dimer models. a. AF2 model of CXCR4 dimer (pink) superimposed onto QUESTS open dimer model (cyan). RMSD=2.4 Å over 549 residues. b. AF2 model of μOR dimer (pink) superimposed onto QUESTS open dimer model (cyan). RMSD=4.1 Å over 556 residues. c. AF2 model of V2R dimer.

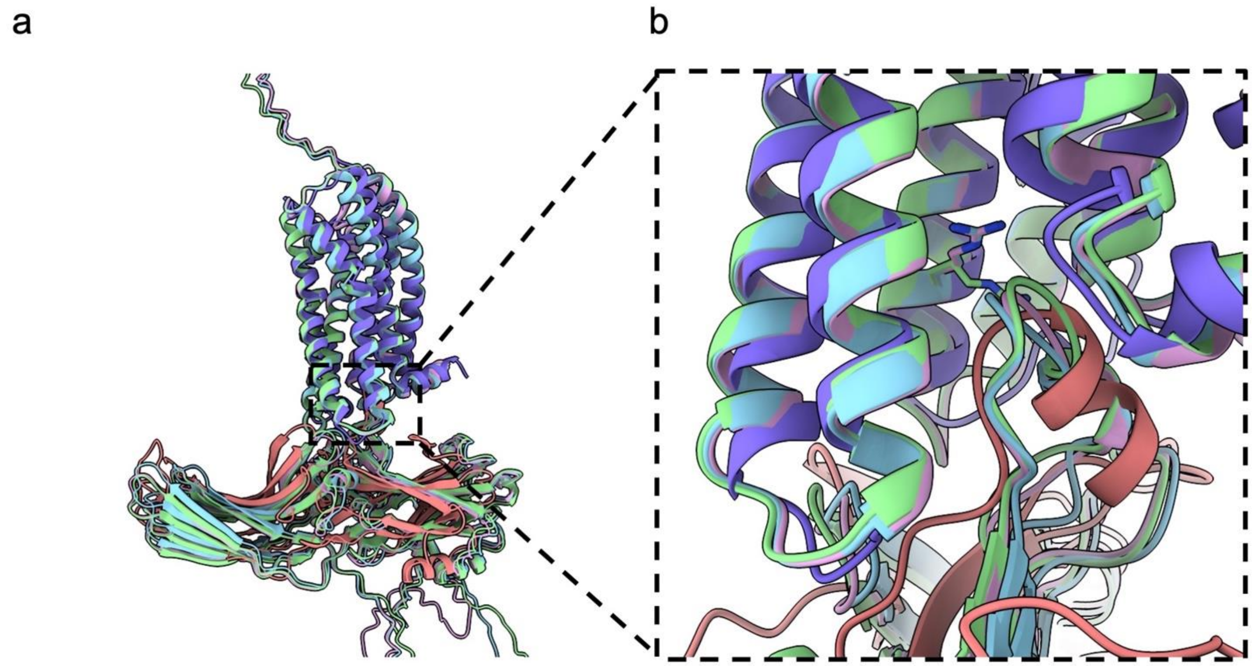

Supplementary Fig. 14. Comparison between AF2 and QUESTS predicted beta-arrestin bound CXCR4 models. a. Top 3 AF2 models (green, cyan, pink) superimposed onto QUESTS model (CXCR4: blue; β-arrestin: salmon). b. zoom on the β-arrestin finger loop binding site.

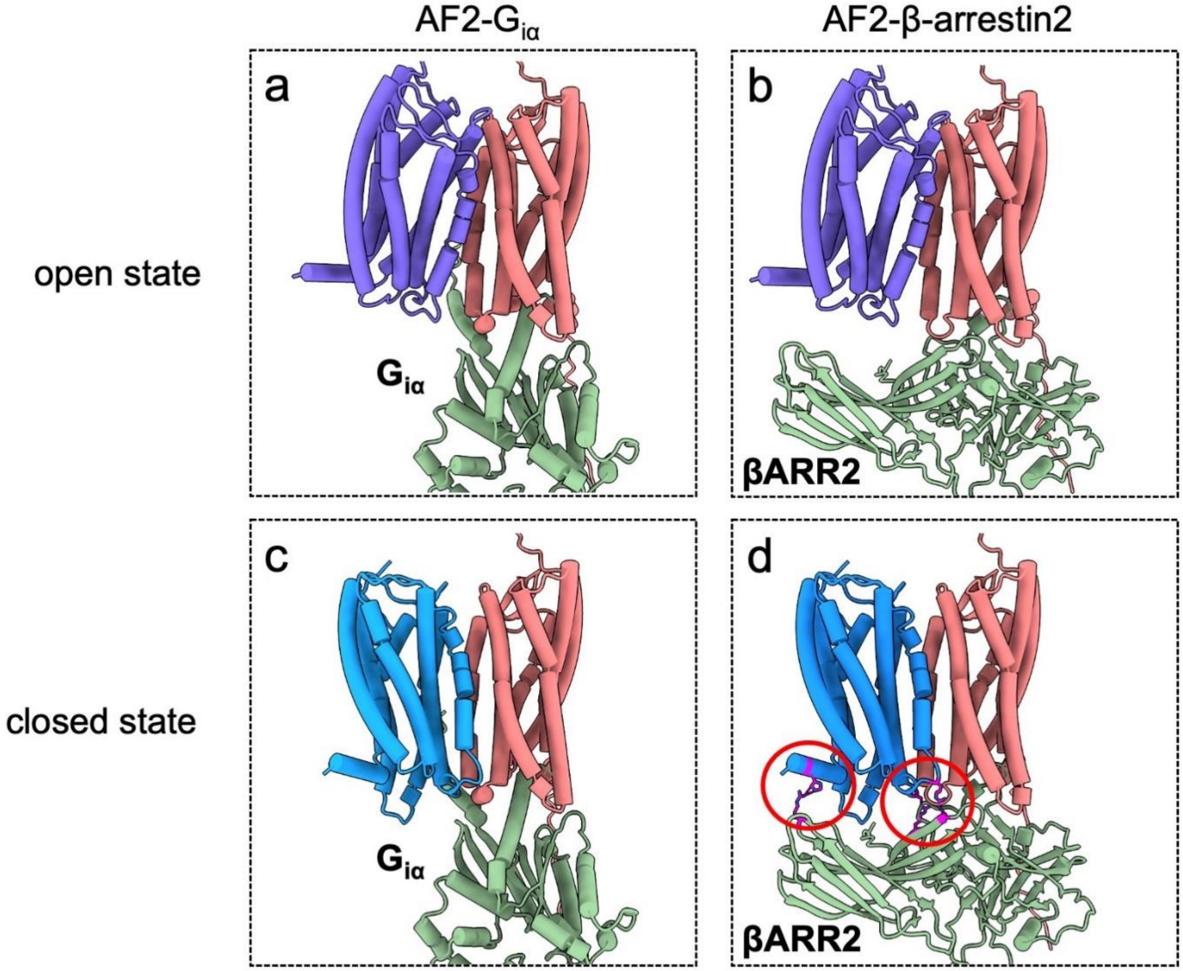

Supplementary Fig. 15. AF2 models predict steric hindrance between β-arrestin and the CXCR4 closed dimer conformation. a. AF2 model of Gi bound CXCR4 monomer (receptor: salmon, Gi: green) aligned to one protomer of the CXCR4 open dimer conformation (blue). b. AF2 model of β-arrestin bound CXCR4 monomer (receptor: salmon, β-arrestin: green) aligned to one protomer of the CXCR4 open dimer conformation (blue). c. AF2 model of Gi bound CXCR4 monomer (receptor: salmon, Gi: green) aligned to one protomer of the CXCR4 closed dimer conformation (blue). d. AF2 model of β-arrestin bound CXCR4 monomer (receptor: salmon, β-arrestin: green) aligned to one protomer of the CXCR4 closed dimer conformation (blue). Potential steric clashes highlighted in red circles were identified using the ChimeraX program[2]. ChimeraX did not identify any clashes in the Gi-bound CXCR4 closed dimer (c).

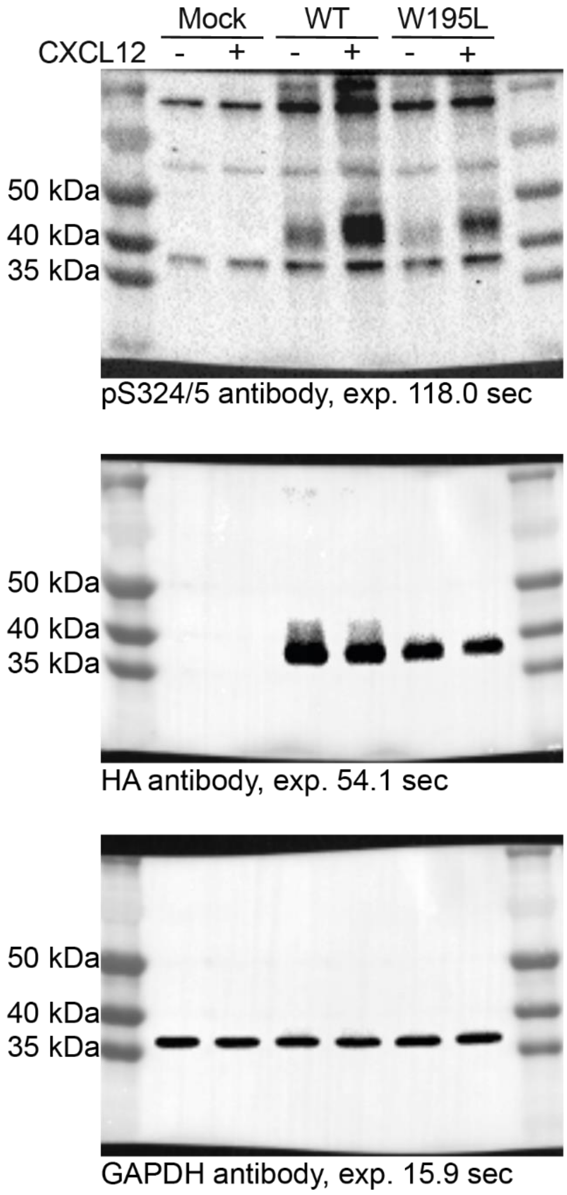

Supplementary Fig. 16. Unprocessed scans of the Western blots presented in Fig. 4b, left panel.

Supplementary References

1. Feng, X., Ambia, J., Chen, K.M., Young, M. & Barth, P. Computational design of ligand-binding membrane receptors with high selectivity. Nat Chem Biol 13, 715-723 (2017).
2. Pettersen EF, Goddard TD, Huang CC, Meng EC, Couch GS, Croll TI, Morris JH, Ferrin TE. UCSF ChimeraX: Structure visualization for researchers, educators, and developers. Protein Sci 30, 70-82 (2021)
