# Supplementary material for: Computationally designed GPCR quaternary structures bias signaling pathway activation
Source: Nat Commun. 2022 Nov 11;13:6826. doi: 10.1038/s41467-022-34382-7 (PMC9652377; doi:10.1038/s41467-022-34382-7)
Supplement: Supplementary file 3 — Reporting Summary [file 41467_2022_34382_MOESM3_ESM.pdf]

nature research

Corresponding author(s): Michel Bouvier

Last updated by author(s): Oct 4, 2022

Reporting Summary

Nature Research wishes to improve the reproducibility of the work that we publish. This form provides structure for consistency and transparency in reporting. For further information on Nature Research policies, see Authors & Referees and the Editorial Policy Checklist.

Statistics

For all statistical analyses, confirm that the following items are present in the figure legend, table legend, main text, or Methods section.

n/a	Confirmed	
☐	☒	The exact sample size (*n*) for each experimental group/condition, given as a discrete number and unit of measurement
☐	☒	A statement on whether measurements were taken from distinct samples or whether the same sample was measured repeatedly
☐	☒	The statistical test(s) used AND whether they are one- or two-sided<br>*Only common tests should be described solely by name; describe more complex techniques in the Methods section.*
☒	☐	A description of all covariates tested
☒	☐	A description of any assumptions or corrections, such as tests of normality and adjustment for multiple comparisons
☐	☒	A full description of the statistical parameters including central tendency (e.g. means) or other basic estimates (e.g. regression coefficient) AND variation (e.g. standard deviation) or associated estimates of uncertainty (e.g. confidence intervals)
☐	☒	For null hypothesis testing, the test statistic (e.g. *F*, *t*, *r*) with confidence intervals, effect sizes, degrees of freedom and *P* value noted<br>*Give P values as exact values whenever suitable.*
☒	☐	For Bayesian analysis, information on the choice of priors and Markov chain Monte Carlo settings
☒	☐	For hierarchical and complex designs, identification of the appropriate level for tests and full reporting of outcomes
☒	☐	Estimates of effect sizes (e.g. Cohen's *d*, Pearson's *r*), indicating how they were calculated

Our web collection on statistics for biologists contains articles on many of the points above.

Software and code

Policy information about availability of computer code

Data collection	BRET, luminescence and HTRF were measured on Mithras LB940, SpectraMax L, Tecan GENios Pro, respectively. Computational data were generated using protein modeling, docking and design applications (IPHoLD, RosettaMembrane) available in the latest version of the Rosetta software package (https://www.rosettacommons.org/software). Complex structures were also modeled using Alphafold2 (https://github.com/sokrypton/ColabFold). Examples with commands/inputs/outputs/code for running the symmetry docking, clustering analysis, Gi & b-arresting docking and Alphafold are provided in the github repository: https://github.com/barth-lab/QUESTS.
Data analysis	In vitro assays were analysed using GraphPad Prism (version 9). Computational data were analyzed using standard clustering programs available in the Rosetta software package (https://www.rosettacommons.org/software) or the hdbscan-clustering program available at https://github.com/scikit-learn-contrib/hdbscan. Structures were analyzed for steric clashes using the ChimeraX software (https://www.cgl.ucsf.edu/chimerax/)

For manuscripts utilizing custom algorithms or software that are central to the research but not yet described in published literature, software must be made available to editors/reviewers. We strongly encourage code deposition in a community repository (e.g. GitHub). See the Nature Research guidelines for submitting code & software for further information.

Data

Policy information about availability of data

All manuscripts must include a data availability statement. This statement should provide the following information, where applicable:

- Accession codes, unique identifiers, or web links for publicly available datasets
- A list of figures that have associated raw data
- A description of any restrictions on data availability

Data availability: The authors declare that all data supporting the findings in this study are presented within the article and its Supplementary Information files.
Code availability: Examples with commands/inputs/outputs/code for running the symmetry docking, clustering analysis, Gi & β-arresting docking and Alphafold are provided in the github repository: https://github.com/barth-lab/QUESTS. The following PDB hyperlinks were also used in the study: 33ODU: 10.2210/pdb3ODU/pdb,

4XT1: 10.2210/pdb4XT1/pdb, 6DDF: 10.2210/pdb6DDF/pdb, 7DW9: 10.2210/pdb7DW9/pdb, 4ZWJ: 10.2210/pdb4ZWJ/pdb, 3SN6: 10.2210/pdb3SN6/pdb and 1GIA: 10.2210/pdb1GIA/pdb.

Field-specific reporting

Please select the one below that is the best fit for your research. If you are not sure, read the appropriate sections before making your selection.

☒ Life sciences ☐ Behavioural & social sciences ☐ Ecological, evolutionary & environmental sciences

For a reference copy of the document with all sections, see nature.com/documents/nr-reporting-summary-flat.pdf

Life sciences study design

All studies must disclose on these points even when the disclosure is negative.

Sample size	Data represent at least three (3) independent experiment performed in triplicate. Based on previous experience and given the extent of variability, sample size of 3 independent experiment is sufficient to observe statistically significant differences. This is a generally accepted approach by the scientific community for this type of data.
Data exclusions	No data were excluded from the analysis
Replication	For each independent experiment, technical triplicates were carried out. At least 3 independent experiments were carried out for all data presented.
Randomization	No samples/organisms/participants were used in this study
Blinding	No samples/organisms/participants were used in this study, so no blinding was possible

Reporting for specific materials, systems and methods

We require information from authors about some types of materials, experimental systems and methods used in many studies. Here, indicate whether each material, system or method listed is relevant to your study. If you are not sure if a list item applies to your research, read the appropriate section before selecting a response.

Materials & experimental systems

n/a	Involved in the study	
☐	☒	Antibodies
☐	☒	Eukaryotic cell lines
☒	☐	Palaeontology
☒	☐	Animals and other organisms
☒	☐	Human research participants
☒	☐	Clinical data

Methods

n/a	Involved in the study	
☒	☐	ChIP-seq
☒	☐	Flow cytometry
☒	☐	MRI-based neuroimaging

Antibodies

Antibodies used	To perform "cell surface ELISA" to monitor WT and mutant HA-CXCR4, HA-CXCR4-YFP and myc-CXCR4-Rluc, anti-HA mouse monoclonal antibody (Santacruz, sc-5759, clone 12CA5) or Myc rabbit monoclonal antibody (Cell Signaling, 2278, clone 71D10) were used, along with Horseradish Peroxidase (HRP)-tagged sheep anti-mouse IgG antibody (GE healthcare, NA931, lot 1523046) or HRP-tagged donkey anti-rabbit IgG antibody (GE healthcare, NA934). To select U87.MG cells stably expressing CXCR4 WT and W195L mutant, Alexa Fluor 488-labeled anti-HA.11 Epitope Tag mouse antibody (Biolegend, clone 16B12) was used. To perform phosphorylation assays on CXCR4, pS324/pS325-CXCR4 (phospho-CXC Chemokine Receptor 4) rabbit antibody (7TM antibodies, 7TM0071A, lot 12190071A001), rat monoclonal anti-HA (Roche, ROAHAMA, clone 3F10) and anti-GAPDH rabbit monoclonal Ab (Cell Signaling, 2118, clone 14C10) were used, along with HRP-tagged donkey anti-rabbit IgG antibody (GE healthcare, NA934) or HRP-tagged goat anti-rat IgG antibody (Sigma, AP136P). To monitor ERK phosphorylation, Advanced Homogenous Time-Resolved FRET (HTRF)-based Phospho-ERK (Thr202/Tyr204) cellular kit (Cisbio, 64AERPET) was used. To monitor CXCR4 expression on the surface of HEK and U87 cells, a human CXCR4 phycoerythrin (PE)-conjugated mouse monoclonal antibody (R&D Systems, FAB170P, clone 12G5) was used.
Validation	Most antibodies used have been validated by the company using either Western blot, Flow cytometry or Immunofluorescence. Furthermore, a negative control of cells transfected with an empty plasmid was also used for a negative background signal.

Eukaryotic cell lines

Policy information about cell lines

Cell line source(s)	HEK293T are derived from HEK293 human embryonic kidney cells containing the SV40 large T antigen, which enables them to produce recombinant proteins within plasmid vectors containing the SV40 promoter. These cells were obtained from ATCC. U87MG cells were obtained from Dr. Bruce Chesebro through the NIH AIDS Reagent Program, Division of AIDS, NIAID, NIH. (Ponten J, MacIntyre EH. Long-term culture of normal and neoplastic human glia. Acta Pathol Microbiol 74:465-486, 1968) (https://www.aidsreagent.org/reagentdetail.cfm?t=cell_lines&id=324).
Authentication	HEK293T cells have been autentified using STR genotyping by Gene Copoeia.
Mycoplasma contamination	Cells were regularly tested for mycoplasma contamination (PCR Mycoplasma Detection kit, abm). If they were testing positive, cells were discarded and restarted from a frozen mycoplasma free cells stock of lower passage. A statement to this effect has been added to the Method of the manuscript.
Commonly misidentified lines (See ICLAC register)	No commonly misidentified cell lines were used in the study.
